# APACHE-II score for anti-tuberculosis tolerance in critically ill patients: a retrospective study

**DOI:** 10.1186/s12879-019-3751-7

**Published:** 2019-02-04

**Authors:** Junke Qiu, Caihong Wang, Xiaohong Pan, Lei Pan, Xiaoqing Huang, Jiekun Xu, Xiaobo Ji, Minjie Mao

**Affiliations:** Department of Tuberculosis Intensive Care Unit, Tuberculosis Diagnosis and Treatment Center of Zhejiang Province, Hang Zhou Red Cross Hospital, Hang Zhou, 310003 China

**Keywords:** Tuberculosis, Critical illness, APACHE-II, Drug tolerance

## Abstract

**Background:**

To investigate the status of anti-tuberculosis treatment in critically ill patients, and to explore the value of APACHE-II score in guiding anti-tuberculosis treatment.

**Methods:**

This analysis included critically ill patients with tuberculosis. The utility of APACHE-II score for predicting drug withdrawal was evaluated using receiver operating characteristic (ROC) curve analysis.

**Results:**

Among 320 patients enrolled (58 ± 22 years; 256 males), 147 (45.9%) had drugs withdrawn. The drug withdrawal group had higher APACHE-II score (median [interquartile range]: 21 [3–52] vs. 17 [4–42] points), higher CD4%, lower hemoglobin level, higher rates of chronic obstructive pulmonary disease (COPD) and chronic renal failure, and lower rate of extrapulmonary tuberculosis (*P* < 0.05). Logistic regression identified APACHE-II score > 18 (odds ratio [95% confidence interval]: 2.099 [1.321–3.334], *P* < 0.01), COPD (1.913 [1.028–3.561], *P* < 0.05) and hemoglobin level (0.987 [0.977–0.997], *P* < 0.05) as independent factors associated with drug withdrawal. At an optimal cutoff of 18.5, the sensitivity, specificity, positive predictive value and negative predictive value of APACHE-II score for predicting drug withdrawal was 59.2, 61.8, 56.9 and 64.1%, respectively.

**Conclusions:**

APACHE-II score > 18 points might predict patient tolerance of anti-tuberculosis treatment.

## Background

Tuberculosis is a global public health problem and the leading cause of death due to infection [1]. In 2016, there were 10.4 million incident cases of tuberculosis and 1.7 million deaths due to the disease [1]. Although the prevalence of smear-positive tuberculosis in China fell from 170 per 100,000 people in 1990 to 59 per 100,000 people in 2010 [2], the burden remains high. Indeed, China has the third-highest incidence of tuberculosis after India and Indonesia [1]. Medical advances, lifestyle changes and improvements in socioeconomic factors have resulted in people in China living longer than before [3], but with poorer physical and mental health than their counterparts 10 years ago [4]. Furthermore, the prolongation of lifespan has led to an increase in the number of elderly people with tuberculosis.

Tuberculosis can potentially be cured with appropriate medication in those with a timely diagnosis [1]. However, elderly patients with tuberculosis often have comorbidities, organ dysfunction or organ failure, which complicates the successful management of the infection. In particular, elderly patients with tuberculosis tend to have more extensive pulmonary lesions, higher sputum positivity, a higher susceptibility to the adverse effects of anti-tuberculosis drugs (including hepatic dysfunction) and higher mortality [5–8]. Elderly patients with tuberculosis have a lower completion rate than younger patients [9, 10] and are considered by some healthcare workers to be an “obstacle” to the control of tuberculosis in China [11]. Thus, the treatment of elderly patients who are critically ill with tuberculosis is highly challenging.

Non-adherence to anti-tuberculosis therapy remains a major issue, and knowledge of the factors that affect treatment completion would enable healthcare providers to implement appropriate strategies to improve adherence. A wide range of factors have been suggested to be associated with non-adherence to treatment for tuberculosis, including male gender, age > 65 years, drug adverse effects, economic factors (including transportation costs), inadequate knowledge about tuberculosis and its therapy, lack of social support, poor relationship/communication with healthcare providers, feeling unwell, tobacco smoking and co-infection with human immunodeficiency virus (HIV) [12–20]. However, to the best of our knowledge, no studies have investigated the factors influencing the tolerability of anti-tuberculosis therapy in patients who are critically ill with tuberculosis.

We hypothesized that comorbidities and APACHE-II score, a disease severity score widely used in the intensive care unit (ICU) [21], might be predictors of the tolerability of anti-tuberculosis therapy in critically ill patients with tuberculosis. Therefore, the aim of this retrospective analysis was to investigate the factors associated with the tolerability of anti-tuberculosis medications in patients with tuberculosis being treated at an ICU in China. It was envisaged that the findings might provide guidance regarding the selection of the anti-tuberculosis regimen in critically ill patients with tuberculosis.

## Methods

### Study design and participants

Critically ill patients with tuberculosis at the Department of Tuberculosis Intensive Care Unit, Hang Zhou Red Cross Hospital between March 2009 and May 2017 were retrospectively analyzed. The inclusion criteria were: 1) diagnosis of tuberculosis [22]; 2) failure of one or more organs, such as respiratory, heart, renal and/or liver failure; 3) admitted to the ICU ≥3 days; and 4) anti-tuberculosis treatment ≥3 days. The exclusion criteria were: 1) non-tuberculous mycobacterial disease; and 2) incomplete clinical data.

The Clinical Ethics Committee of Hangzhou Red Cross Hospital approved the study. Informed consent was waived due to the retrospective study design.

### Data collection

Data including age, gender, clinical characteristics of the tuberculosis episode, comorbidities and other relevant clinical factors, blood tests, Sequential Organ Failure Assessment (SOFA) score [23], APACHE-II score [21], anti-tuberculosis treatment regimen used, drug therapy, drug withdrawal, and adverse reactions (based on the toxicity and allergy reporting system implemented in Chinese hospitals) were recorded. Patients who had discontinued at least one anti-tuberculosis drug during therapy were included in the drug withdrawal group.

### Statistical analysis

Data were analyzed using SPSS 16.0 (IBM, USA). Normally distributed data are presented as the mean ± standard deviation and compared using Student’s t-test. Non-normally distributed data are expressed as median (interquartile range) and compared using the Wilcoxon rank sum test. A two-sided *P*-value < 0.05 was indicated statistical significance. Differences in the underlying diseases were assessed with the χ^2^ test. Multivariable logistic regression analysis with forward selection was used to identify factors associated with anti-tuberculosis tolerance; factors with *P* values < 0.05 in univariable analyses were considered for inclusion in the multivariable analysis. Receiver operating characteristic (ROC) curve analysis was used to evaluate APACHE-II score for predicting the discontinuation of drug therapy.

## Results

### Demographic and clinical characteristics of the study participants

A total of 320 patients (age, 58 ± 22 years; 256 males, 80.0%) were included in the analysis, with 147 (45.9%) in the drug withdrawal group and 173 (54.1%) in the therapy completion group. The demographic and clinical characteristics of the patients in the two groups are presented in Table 1. Compared with the therapy completion group, the drug withdrawal group had a significantly higher APACHE-II score (within 24 h after admission to the ICU) and CD4% (i.e. percentage of lymphocytes that are CD4-positive), a significantly lower hemoglobin level, significantly higher frequencies of chronic obstructive pulmonary disease (COPD) and chronic renal failure as comorbidities, and a significantly lower rate of extrapulmonary tuberculosis (all *P* < 0.05; Table 1). A total of 110 patients were using vasoactive drugs. However, there were no significant differences between groups in any of the other parameters assessed (see Table 1 for details).

**Table 1:** Demographic and clinical characteristics of the study participants

Parameter	Drug withdrawal group (*n* = 147)	Therapy completion group (*n* = 173)	*P*
*Demographic characteristics*			
Age (years)	65 (2–94)	62 (17–90)	0.24
Male	118 (80.3%)	138 (79.8%)	0.91
*Tuberculosis characteristics*			
Pulmonary tuberculosis	131 (89.1%)	153 (88.4%)	0.85
Sputum smear positive	98 (66.7%)	105 (60.7%)	0.27
Extrapulmonary tuberculosis	51 (34.7%)	83 (48.05)	0.02
First treatment	116 (78.9%)	145 (83.8%)	0.26
*Comorbidities*			
Diabetes mellitus	26 (17.7%)	32 (18.5%)	0.85
Hypertension	42 (28.6%)	40 (23.1%)	0.27
Coronary heart disease	23 (15.6%)	17 (9.8%)	0.12
Chronic heart failure	37 (25.2%)	33 (19.1%)	0.19
Atrial fibrillation	12 (8.2%)	9 (5.2%)	0.29
Chronic renal failure	14 (9.5%)	7 (4.0%)	0.049
Lung cancer	3 (2.0%)	5 (2.9%)	0.63
Other malignant tumors	12 (8.2%)	10 (5.8%)	0.40
COPD	31 (21.1%)	21 (12.1%)	0.03
Bronchiectasis	5 (3.4%)	4 (2.3%)	0.56
Autoimmune disease	12 (8.2%)	15 (8.7%)	0.87
Neurological disorders	20 (13.6%)	18 (10.4%)	0.38
Viral hepatitis B	7 (4.8%)	14 (8.1%)	0.23
*Previous medical interventions*			
Long-term steroids	12 (8.2%)	12 (6.9%)	0.68
Long-term immunosuppressants	2 (1.4%)	4 (2.3%)	0.53
Mechanical ventilation	119 (81.0%)	124 (71.7%)	0.053
*Blood test results*			
White blood cell (10^9^/L)	9.8 (1.5–35.5)	10.4 (0.4–41.2)	0.27
Platelet (10^9^/L)	158 (2–576)	193 (12–723)	0.06
Hemoglobin (g/L)	92 (41–150)	104 (38–160)	0.001
Total bilirubin (μmol/L)	12.1 (2.5–269.6)	12.9 (1–92.2)	0.40
Direct bilirubin (μmol/L)	5.8 (0.1–131.4)	6.6 (0.1–65.1)	0.24
Indirect bilirubin (μmol/L)	5.1 (0.3–138.2)	5.4 (0.1–49)	0.54
Uric acid (μmol/L)	209 (29–1102)	225 (1–831)	0.21
Urea nitrogen (mmol/L)	6.4 (0.9–34.6)	5.9 (1.4–75.8)	0.07
Total protein (g/L)	57.3 (26–83)	56.7 (30.1–84.3)	0.62
Albumin (g/L)	27.3 (10.6–47.4)	28.0 (15.4–47.0)	0.15
Globulin (g/L)	28.4 (15.4–46)	27.3 (10.7–50.6)	0.08
Aspartate aminotransferase (U/L)	33 (7–1241)	28 (6–2971)	0.21
Alanine aminotransferase (U/L)	19 (3–461)	22 (2–757)	0.23
Alkaline phosphatase (U/L)	94 (28–393)	84 (25–927)	0.12
Gamma-glutamyltransferase (U/L)	49 (10–813)	44 (10–991)	0.92
Prealbumin (mg/L)	86 (4–272)	105 (8–394)	0.26
Retinol binding protein (mg/L)	12.0 (1.1–73.8)	12.3 (0.5–66.0)	0.51
Fructosamine (mmol/L)	1.47 (0.70–3.06)	1.46 (0.81–4.02)	0.62
Immunoglobulin A (g/L)	2.96 (0.50–8.82)	2.75 (0.22–6.15)	0.49
Immunoglobulin G (g/L)	12.5 (4.4–24.5)	11.6 (3.0–25.8)	0.19
Immunoglobulin M (g/L)	1.00 (0.35–3.39)	0.93 (0.28–3.52)	0.60
Complement component C3 (g/L)	0.77 (0.08–1.64)	0.89 (0.20–2.51)	0.15
Complement component C4 (g/L)	0.24 (0.02–0.47)	0.25 (0.07–0.64)	0.40
Cancer antigen CA-125 (kU/L)	86.0 (7.4–1977.2)	74.3 (3.3–1241.5)	0.54
CD3 (%)	63.0 (35.6–94.1)	57.9 (26.3–90.5)	0.09
CD4 (%)	33.6 (4.61–64.5)	29.4 (6.67–73.1)	0.02
CD8 (%)	22.3 (3.3–56.9)	22.0 (4.66–76.2)	0.81
CD4/CD8 ratio	1.54 (0.25–13.58)	1.4 (0.13–11.01)	0.23
CD19 (%)	9.3 (0.1–46.3)	10.2 (0.3–48.3)	0.27
CD16 and CD56 (%)	11.0 (0.17–43.6)	11.5 (0.1–60.5)	0.36
*Illness severity scales*			
APACHE-II score	21 (3–52)	17 (4–42)	< 0.001
SOFA score	6 (0–17)	5 (0–18)	0.10

Data are presented as median (interquartile range) or *n* (%). *COPD* chronic obstructive pulmonary disease, *SOFA* Sequential Organ Failure Assessment

### Anti-tuberculosis treatment regimens

A total of 320 patients received anti-tuberculosis treatment. Information regarding the anti-tuberculosis drug regimens used in patients from the two groups is shown in Table 2. An HRZE regimen (isoniazid [H], rifampicin [R], pyrazinamide [Z] and ethambutol [E]) was used in the majority of patients (61.2% in the drug discontinuation group and 60.1% in the therapy completion group), and over 90% of patients received at least 3 anti-tuberculosis drugs (94.6% in the drug discontinuation group and 90.2% in the therapy completion group). There were no significant differences between groups in the treatment regimen used (Table 2). Among those patients, there were six patients with streptomycin (s) resistance, two with R resistance, four with HRS resistance, one with HRES resistance, five with HR resistance, and nine with H resistance. There were 43 patients without susceptibility results.

**Table 2:** Comparison of treatment regimens between groups

Treatment characteristic	Drug withdrawal group (*n* = 147)	Therapy completion group (*n* = 173)	*P*
*Isoniazid (H) and rifampicin (R)*			0.27
Neither H nor R used	6 (4.1%)	15 (8.7%)	
H used but R not used	48 (32.7%)	54 (31.2%)	
Both H and R used	90 (61.2%)	104 (60.1%)	
*Use of HRZE regimen*			0.84
HRZE regimen used	90 (61.2%)	104 (60.1%)	
Non-HRZE regimen used	57 (38.8%)	69 (39.9%)	
*Number of drugs used*			0.21
≤ 2	8 (5.4%)	17 (9.8%)	
3	41 (27.9%)	49 (28.3%)	
4	57 (38.8%)	73 (42.2%)	
5–6	41 (27.9%)	34 (19.7%)	

Data are presented as *n* (%). HRZE: isoniazid (H), rifampicin (R), pyrazinamide (Z) and ethambutol (E)

### Frequency of withdrawal and reasons for withdrawal of anti-tuberculosis drugs

The rates of withdrawal of each anti-tuberculosis drug are presented in Table 3. The most frequently withdrawn drugs were rifapentine (41.7%) and rifampicin (41.6%) followed by pyrazinamide (29.6%), isoniazid (25.9%) and ethambutol (16.3%). As detailed in Table 4, the most common reasons for drug withdrawal were liver dysfunction (42.5%), drug allergy (15.0%), thrombocytopenia (15.0%), disease deterioration (7.2%) and renal dysfunction (6.0%).

**Table 3:** Frequencies of anti-tuberculosis drug use and withdrawal

Drug	Use of drug (*n*)	Withdrawal of drug (*n*)	Rate of withdrawal (%)
Isoniazid	293	76	25.9%
Rifampicin	197	82	41.6%
Ethambutol	258	42	16.3%
Pyrazinamide	135	40	29.6%
Quinolones	217	24	11.1%
Rifapentine	60	25	41.7%
Others	55	8	14.6%

**Table 4:** Reasons for withdrawal of anti-tuberculosis drugs

Reason for drug withdrawal	*n*	%
Liver dysfunction	71	42.5%
Drug allergy	25	15.0%
Thrombocytopenia	25	15.0%
Disease deterioration	12	7.2%
Renal dysfunction	10	6.0%
Use of voriconazole	7	4.2%
Convulsions	4	2.4%
Gastrointestinal bleeding	2	1.2%
Economic reasons	2	1.2%
Based on resistance to the drug	1	0.6%
Malignant arrhythmia	1	0.6%
Psychiatric symptoms	1	0.6%
Unclear	6	3.6%
Total	167	100.0%

### Survival

In the therapy completion group, 112 patients were transferred to a hospital ward and 61 patients died. In the drug withdrawal group, 62 patients were transferred to a hospital ward and 85 patients died. The overall ICU mortality rate was 45.6%, and the ICU mortality rate was significantly higher in the drug withdrawal group than in the therapy completion group (57.8% vs. 35.3%, *P* < 0.05).

### Logistic regression analysis of factors associated with anti-tuberculosis drug withdrawal

Based on the results of the univariable analysis (Table 1), APACHE-II score (> 18 vs. ≤18), hemoglobin level (analyzed as a continuous variable), COPD (yes vs. no) and chronic renal failure (yes vs. no) were included in the regression analysis. The results showed that APACHE-II score > 18 (OR: 2.10; 95% CI: 1.32–3.33; *P* < 0.01), comorbid COPD (OR: 1.91; 95% CI: 1.03–3.56; *P* < 0.05) and hemoglobin level (OR: 0.99; 95% CI: 0.98–0.997; *P* < 0.05) were independently associated with the withdrawal of anti-tuberculosis treatment (Table 5).

**Table 5:** Logistic regression analysis of factors independently associated with anti-tuberculosis drug withdrawal

Variable	Odds ratio	95% confidence interval	*P*
APACHE-II score > 18 points	2.10	1.32–3.33	0.002
COPD (yes vs. no)	1.91	1.03–3.56	0.04
Hemoglobin level (continuous)	0.99	0.98–0.997	0.01

*COPD* chronic obstructive pulmonary disease

### ROC curve analysis of the ability of APACHE-II score to predict anti-tuberculosis drug withdrawal

**Fig. 1:**
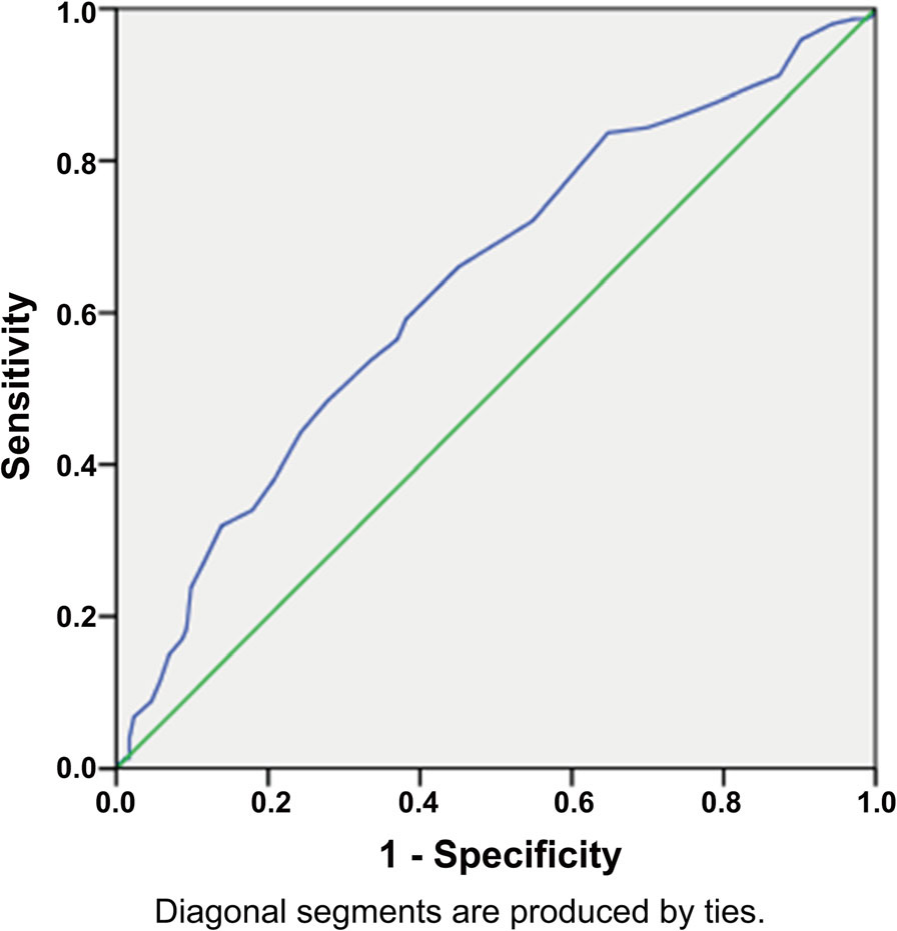
Receiver operating characteristic curve analysis for the prediction of anti-tuberculosis drug withdrawal by APACHE-II score

The area under the ROC curve was 0.64 (Fig. 1), and the Youden index was 0.21. Using an optimal cutoff value of 18.5 points, the sensitivity, specificity, positive predictive value and negative predictive value of APACHE-II score in the prediction of anti-tuberculosis drug withdrawal was 59.2, 61.8, 56.9 and 64.1%, respectively.

## Discussion

A notable finding of the present study was that patients in the drug withdrawal group had higher APACHE-II score, lower hemoglobin level and higher rates of COPD. Furthermore, logistic regression identified an APACHE-II score > 18 points, comorbid COPD and hemoglobin level as independent factors associated with anti-tuberculosis drug withdrawal. In addition, when an optimal cutoff of 18.5 was used, APACHE-II score had reasonable sensitivity, specificity, positive predictive value and negative predictive value in the prediction of drug discontinuation. Taken together, our findings suggest that an APACHE-II score > 18 points might be a useful indicator for predicting patient tolerance of anti-tuberculosis treatment.

Adverse reactions caused by anti-tuberculosis drugs are important factors affecting the success of standard therapy for tuberculosis. Previous studies have reported that the total incidence of adverse reactions caused by anti-tuberculosis drugs is 12.6% in China [24], 12.5% in Portugal [25], 17.1% in Korea [26], and 33.6% in Iran [27]. The apparent variation between studies may be related to differences in the incidence of tuberculosis, treatment strategies used or definitions of adverse reactions, as well as other factors. The incidence of adverse reactions in the present study was substantially higher than the average national incidence in China. This is likely due to the patients in our study being critically ill and thus treated in the Department of Tuberculosis Intensive Care Unit. Critically ill patients are likely to have more comorbidities and more severe disease, which would be expected to make them more susceptible to the development of adverse reactions induced by anti-tuberculosis drugs. To our knowledge, the incidence of adverse reactions to anti-tuberculosis drugs in critically ill patients has not been reported previously, either in China or elsewhere.

Clinical studies have found that the occurrence of adverse reactions to anti-tuberculosis drugs is related to a wide variety of factors, including patient age, patient gender, multidrug-resistance, smoking status, and comorbidities such as HIV co-infection, viral hepatitis and diabetes mellitus [25–30]. In this study, logistic regression analysis identified COPD as an independent factor associated with the withdrawal of anti-tuberculosis treatment in critically ill patients with tuberculosis. Tuberculosis combined with COPD is thought to be the leading cause of death due to respiratory infection [31]. Tuberculosis leads to decreased lung volume and airway stenosis, which are risk factors for the occurrence of COPD, while long-term use of steroids for COPD leads to decreased immune function, which is a risk factor for tuberculosis. Thus, there is an association between tuberculosis and COPD [31, 32]. In this study, the number of patients with COPD and the mortality rate were both significantly higher in the drug withdrawal group than in the therapy completion group, which would be consistent with comorbid COPD increasing the risk of death in patients with tuberculosis, as has been reported previously [33, 34].

The APACHE-II scoring system is the most commonly used method for assessing the severity of critical illness, with a higher score indicating more severe disease. APACHE-II score has been reported to be an independent factor associated with the prognosis of patients with ARDS caused by tuberculosis [35–39]. In the present study, patients in the drug withdrawal group had a significantly higher APACHE-II score than patients in the therapy completion group, and an APACHE-II score > 18 points was an independent factor associated with anti-tuberculosis drug withdrawal in critically ill patients with tuberculosis. Therefore, we suggest that the APACHE-II score may have certain clinical value for predicting whether a critically ill patient with tuberculosis will tolerate anti-tuberculosis treatment.

Patients in the drug withdrawal group had a significantly lower level of hemoglobin on admission than those in the therapy completion group, and a lower hemoglobin level was an independent factor associated with anti-tuberculosis drug withdrawal. Tuberculosis-associated anemia is a recognized phenomenon but is usually mild and resolves following successful pharmacologic therapy [40]. However, anti-tuberculosis drugs can cause hematologic abnormalities, including anemia, during intensive therapy [41]. It is possible that patients with a lower baseline hemoglobin level before the initiation of treatment are more susceptible to reductions in hemoglobin level caused by therapy, which in turn would make drug withdrawal due to the manifestation of adverse events more likely. Another possibility is that low hemoglobin levels impair the effectiveness of anti-tuberculosis therapy. Consistent with this possibility, anemia was found to be associated with delayed sputum conversion after therapy, and it was suggested that the impaired response to treatment in those with low hemoglobin levels may be due to malnourishment and impaired T cell immunity secondary to zinc and iron deficiency [42]. Further research is needed to clarify the mechanism underlying the association between pre-therapy hemoglobin level and subsequent drug withdrawal.

In this study, abnormalities of liver and kidney function, thrombocytopenia and disease deterioration accounted for around 70% of cases of drug withdrawal. Hepatic injury was the most common adverse reaction caused by anti-tuberculosis drugs, as described in other publications [24]. The incidence reported in previous studies vary greatly, from 2 to 28% [27], possibly due to differences in the criteria used to evaluate liver dysfunction, population characteristics and treatment regimens. The rate of drug withdrawal due to liver damage in this study was 22.7%, similar to that reported previously [43]. Rifampicin, isoniazid and pyrazinamide are the main anti-tuberculosis drugs that cause liver damage [44], which likely explains why the frequencies of withdrawal of these drugs were higher than those of the other drugs in this study. Allergic reactions are also commonly encountered during the treatment of tuberculosis, and the incidence of allergic reactions in this study was similar to the incidence of 3.4–17.8% reported during standard therapy [45]. The allergic reactions reported in this study were mild and mainly manifested as urticaria and drug fever, with no cases of exfoliative dermatitis or bullous dermatitis. The incidence of thrombocytopenia as an adverse reaction of anti-tuberculosis drugs has been rarely reported, although it has been mentioned in case reports [46, 47]. In patients with acquired immunodeficiency syndrome (AIDS), the rate of thrombocytopenia as an adverse reaction to anti-tuberculosis medication was 17.3% [48], similar to the incidence observed in the present study. It is worth noting that patients with tuberculosis also have immune dysfunction [49], although the underlying mechanisms need further exploration. Renal dysfunction was a relatively rare adverse reaction in our study, although its incidence was similar to that reported in the literature (7.1%) [50]. Fungal infections cannot be ignored in critically ill patients (since this is known to increase the risk of death), and some patients in this study needed anti-*Aspergillus* treatment with voriconazole. Since rifampicin is an inducer of CYP450, which reduces the blood concentration of voriconazole, it was discontinued during antifungal treatment. In addition, drug withdrawal in some patients was related to a continuous deterioration in their clinical condition, with worsening respiratory failure and hemodynamic instability. The other, less common reasons for drug withdrawal were likely not related to anti-tuberculosis medication and included gastrointestinal bleeding, malignant arrhythmias, psychiatric symptoms and the results of drug susceptibility testing.

This study has some limitations. First, this was a retrospective analysis and so may have been prone to selection bias or information bias. Some data are not very accurate, and some indicators cannot be unified. Indeed, the reasons for treatment discontinuation were not reliably and consistently indicated in the medical charts and could not be used for analysis. In addition, because patients may have been visiting multiple departments or hospitals, the exact timing of drug start and discontinuation in relation to diagnosis could not be analyzed. The height of the patients was not reliably indicated in the charts and body mass index could not be calculated. Second, other possible confounding factors not included in the analysis may have influenced the results. Third, this was a single-center study, so the generalizability of the findings is not known. Fourth, factors associated with prognosis (i.e. survival) were not assessed. Fifth, factors associated with the withdrawal of each individual drug or with each reason for withdrawal were not investigated. We hope to conduct a prospective multicenter study in the future in order to address those issues.

## Conclusions

In conclusion, comorbid COPD, APACHE-II score > 18 points and hemoglobin level were independent factors associated with the withdrawal of anti-tuberculosis drugs in critically ill patients with tuberculosis. In addition to its utility in assessing the severity of critical illness, the APACHE-II score might be a useful indicator for predicting patient tolerance of anti-tuberculosis treatment.

## Abbreviations

COPD: Chronic obstructive pulmonary disease
HIV: Human immunodeficiency virus
ICU: Intensive care unit
ROC: Receiver operating characteristic
SOFA: Sequential Organ Failure Assessment
